# Supplementary material for: Echinochrome A Treatment Alleviates Atopic Dermatitis-like Skin Lesions in NC/Nga Mice via IL-4 and IL-13 Suppression
Source: Mar Drugs. 2021 Nov 1;19(11):622. doi: 10.3390/md19110622 (PMC8625509; doi:10.3390/md19110622)
Supplement: Supplementary file 1 [file marinedrugs-19-00622-s001.zip › marinedrugs-1418953-supplementary.pdf]

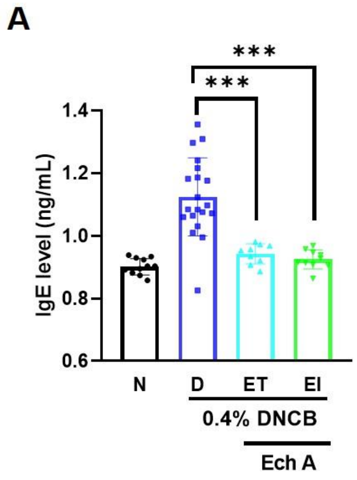

Figure S1. Serum IgE levels of EchA treatment on DNCB-induced AD-like skin lesions in NC/Nga mice. (A) ET and EI groups decreased serum IgE levels compared to D group. The data shown in the graphs represent the mean ± SEM. ***$p < 0.001$.
